# A compact automated titration system, applied to a high-precision determination of calcium in sea-water

**DOI:** 10.1155/S1463924682000212

**Published:** 1982

**Authors:** L. Anderson, A. Granéli

**Affiliations:** 1 Department of Analytical and Marine Chemistry Chalmers University of Technology and University of Gothenburg Göteborg S-412 96 Sweden; 2 Astra Läkemedel AB Södertälje S-151 85 Sweden


Journal of Automatic Chemistry, Volume 4, Number 2 (April–June 1982), pages 75–78

### A compact automated titration system, applied to a high-precision determination of calcium in sea-water

**L. Anderson and A. Granéli***

*Department of Analytical and Marine Chemistry, Chalmers University of Technology and University of Gothenburg, S-412 96 Göteborg, Sweden*

### Introduction

The personal computer, a consumer product of the microprocessor industry, can now be used to control chemical experiments which some years ago required a fairly expensive minicomputer [1]. Whether a mini- or microcomputer is used in an experiment, it must be able to communicate with external devices such as analogue-to-digital converters (ADC), digital multimeters (DMM), digital-to-analogue converters (DAC) and relays. This may be done by using ordinary parallel input/output (I/O) interfaces, or by employing devices designed to interface directly with the specific computer bus used. However, computers as well as instruments are increasingly being equipped with the so-called General Purpose Instrument Bus (GPIB or IEEE-488 bus). This standardized interface greatly simplifies the inter-connection of computers and instruments and can also simplify programming.

This paper describes a titration system which is controlled by a Commodore PET 2001 personal computer via its I/O port and its IEEE-488 bus. The titration system is adapted for high-precision determination of calcium in sea-water. The small physical size of the set-up makes it most suitable for use where laboratory space is limited, for example on board a research vessel.

### Instrumentation

### *Titration unit*

The unit used for titration is a modified Metrohm motor burette, first described by Granéli and Anfält [2]. The burette (figure 1) acts as a measuring device, a titration vessel and a cuvette. The solenoid valves, connected to the top lid of the burette, are all Angar's Teflon Model 190 (Angar Scientific, 52 Horse Hill Road, Cedar Knolls, New Jersey 07927, USA). The burette's alternating current (a.c.) motor is replaced by a four-phase stepper motor and the up and down movement of the piston is determined by the direction of rotation of the stepper motor.

### *Photometer*

A Brinkman PC/600 Colorimeter is used. The light, which is guided to the burette by fibre optics, is passed through the burette and reflected back to the fibre optics. The wavelength is determined by an interference filter. An HP 3436A digital multimeter is connected to the transmittance output of the colorimeter.

### *Computer*

The Commodore PET 2001 has an 8 k random-access memory, a 14 k read-only memory, and uses a cassette tape for program storage. The programming language is normally BASIC, although it is possible to write subroutines in machine code. For communication with external devices, the PET has an IEEE-488 bus [3] and an 8-bit parallel user port. It is possible to connect up to 15 devices to the IEEE-488 bus, as long as the bus extension from the PET does not exceed 20 m, with a maximum inter-device spacing of 5 m. The eight lines of the parallel user port are individually programmable as inputs or outputs. This is done by setting the corresponding bits in the data direction register—high for output and low for input. The titration system has two devices connected to the bus (see figure 1): a printer, where the results and primary data are reported; and a multimeter, which is connected to the colorimeter. Communication on the bus follows a handshaking procedure. A simplified IEEE-488 handshake timing diagram is shown in figure 2.

When data are transmitted from the multimeter to the PET, the multimeter acts as talker and the PET as listener. When the BASIC command INPUT #... is executed by the PET, it sets the NRFD (not ready for data) signal high (ready for data). If the DAV (data are valid) signal set by the talker does not go low (data valid) in 64 ms after the NRFD signal went high, the PET fails in the handshaking procedure. The multimeter may be working in two modes, either 'Talk Only' or 'Addressable to Talk' mode. The Talk Only mode puts the PET in the timing problem described above. The multimeter needs a trigger signal if it is working in the Addressable to Talk mode. This trigger signal is the ASCII code for GET in the Command mode of operation at the bus communication (ATN true). There is no software support for BASIC communication in the Command mode on the PET IEEE-488 bus.

A BASIC program, where extensive use of the PEEK and POKE commands is necessary for reading the multimeter in the Addressable to Talk mode, is shown in figure 3. Since the I/O modes of the signal DAV have been mixed in the *PET User Manual* published in October 1978 (on table 7.25, p. 88), some hardware addresses in the program do not agree with those in the manual.

The printer used is a Texas Silent terminal with an RS 232 interface. It is connected to the IEEE-488 bus by a TNW 488/232 Serial Interface module, produced by Net Works (the Net Works, 5924 Quiet Slope Drive, San Diego, California 92120, USA). The baud rate is selected by jumpers on the interface and the maximum speed of the printer, 300 baud, is used.

The parallel I/O port has three functions: (1) to drive the stepper motor (two lines); (2) to drive the stirrer (one line); and (3) to control the solenoid valves (three lines). One of the lines to the stepper motor controls the direction of rotation, the other is used to trigger the motor drive logic. The three lines to the valves are decoded so that it is possible to control up to eight valves.

**Present address: Astra Läkemedel AB, S-151 85 Södertälje, Sweden.*

0142 0453/82/0402 0075 $05·00 © 1982 Taylor & Francis Ltd

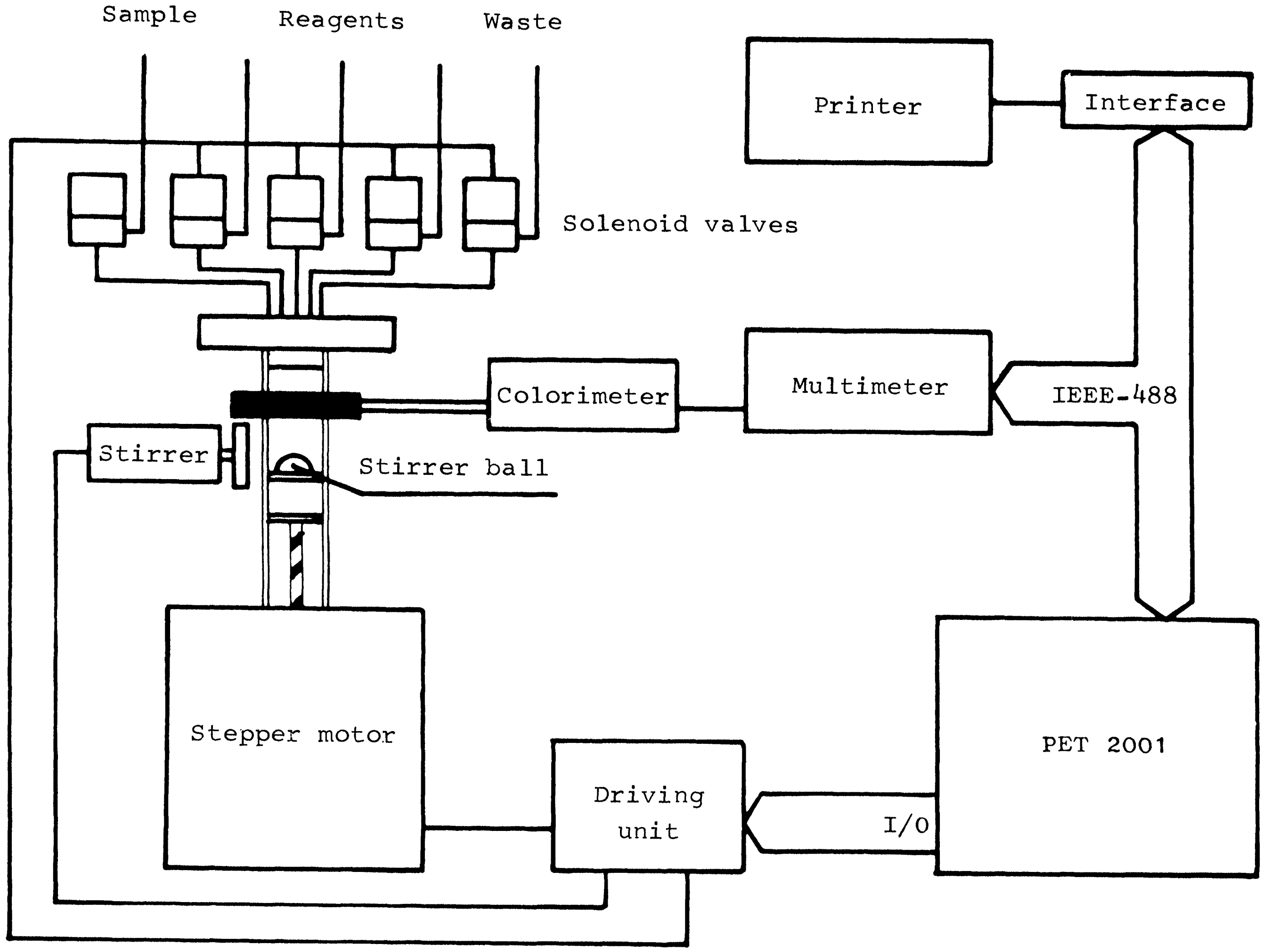

*Figure 1. Schematic diagram of the titration system.*

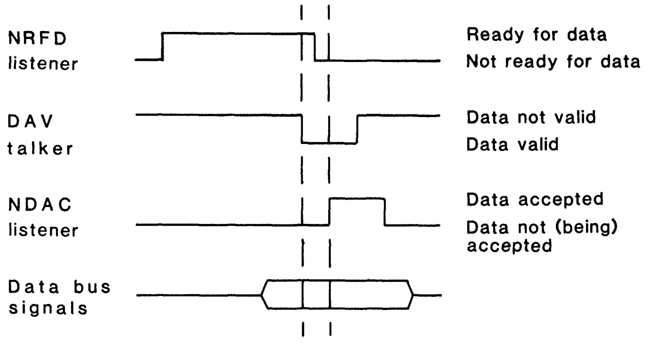

*Figure 2. The IEEE 488 bus's handshaking timing diagram. The ATN (attention) signal determines whether data or commands should be sent on the data bus. Commands are sent and received with the ATN true (low). Abbreviations: NRFD = not ready for data; DAV = data are valid; NDAC = no data accepted.*

| | |
|---|---|
| 1000 OPEN 1,23,1 | Open logical file 1 on device 23. |
| 1005 POKE 59426,247 | Put the ASCII code for the GET command on the data address. |
| 1010 Q=PEEK (59456) | |
| 1020 IF Q AND 2 THEN 1030 | NRFD (input) high? |
| 1025 GO TO 1010 | |
| 1030 POKE 59456,0 | Put ATN low (command mode). |
| 1040 POKE 59427,48 | Put DAV (output) low. |
| 1050 Q=PEEK (59425) | |
| 1060 IF Q AND 8 THEN 1070 | NDAC (input) high? |
| 1065 GO TO 1050 | |
| 1070 POKE 59427,56 | Put DAV (output) high. |
| 1080 PRINT #1,; | Turn off device 23 as listener. |
| 1090 FOR K=1 TO 200:NEXT K | Wait for the multimeter. |
| 1100 INPUT #1,B | Get a multimeter reading. |
| 1110 CLOSE 1 | Close logical file 1. |
| 1120 RETURN | |

*Figure 3. Subroutine for reading the multimeter in 'Addressable to Talk' mode.*

### Experimental

### *Chemical application*

Photometric titration of calcium in the presence of higher concentrations of magnesium, which was described by Jagner [4], has been adapted to the titration system described. Calcium is titrated at pH 8·6 using EGTA as titrant. A small amount of zinc-EGTA is added, and the decrease of calcium is followed by the simultaneous titration of zinc using zincon as indicator. The experimental data for absorbance versus volume of titrant added ($v$) are linearized by means of the Gran function $F$ [5],

$$F = v_1 * (v_0 + v) * (A - A\text{-min})/(A\text{-max} - A)$$

where $v_0$ is the total volume (excluding titrant); $A$, $A$-min, and

$A$-max are absorbance values (620 nm); and

$$v_1 = v + (v_0 + v) * [\mathrm{ZnOHI}]/t_{\mathrm{EGTA}}$$

or

$$v_1 = v + v_0 * [I]_{\mathrm{tot}}/t_{\mathrm{EGTA}} * (A - A\text{-min})/(A\text{-max} - A\text{-min})$$

where $t_{\mathrm{EGTA}}$ is the concentration of titrant and $[I]_{\mathrm{tot}}$ the total concentration of the indicator at volume $v_0$. $v_1$ has been introduced to compensate for the EGTA liberated from the zinc-EGTA complex during the formation of the zinc-indicator complex.

### *Reagents*

A 0·010 M $Na_4EGTA$ solution is prepared from $H_4EGTA$ and NaOH. A borate buffer at pH = 8·6 with an ionic strength of 0·5 M is used. The indicator reagent is a mixture of two

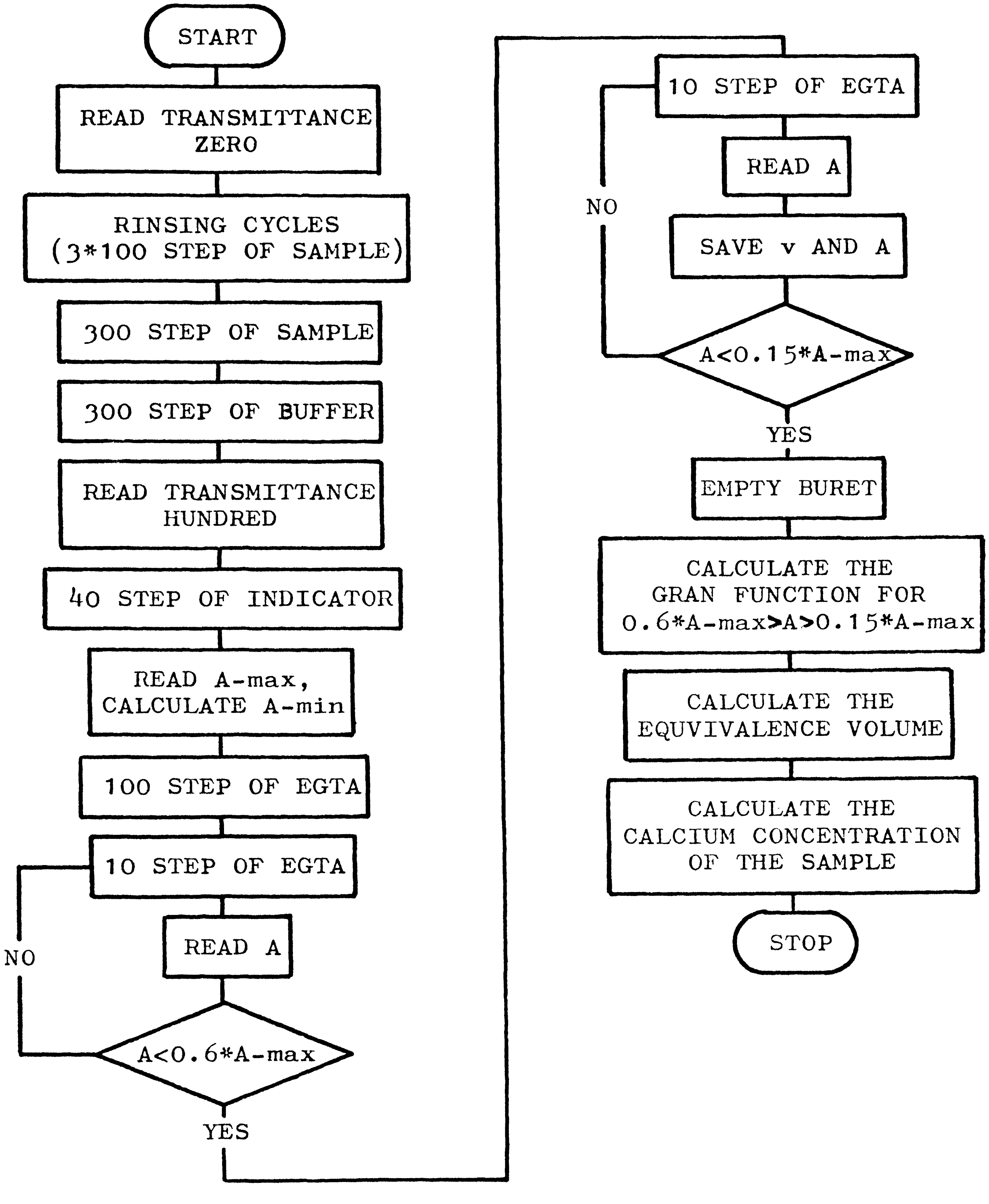

*Figure 4. Flow chart of the titration program.*

solutions: 0·010 M Zn-EGTA in water, and 0·02% zincon in 50% ethanol water. A ratio of about two to three of the two solutions was chosen, this was in order to have all the zincon complexed to zinc at the first titration point.

### Procedure

The five solenoid valves are connected to the different solutions by Teflon tubing. The dynamic flow resistance is held as low as possible to minimize under-pressure when the piston is going down, thus bubble formation is avoided.

The titration procedure is shown in the flow chart in figure 4. The concentration of the indicator reagent is held rather low for two reasons: first, to get a good reproducibility of the absorbance maximum value; second, to avoid a decrease of the transmittance 100% value because of diffusion at the inlet to the burette. The absorbance minimum is determined by an over-titration with $Na_4$EGTA. As the transmittance reading for $A$-min is high, there is some uncertainty about the reading. The ratio of $A$-max/$A$-min is therefore determined in a number of titrations, and a mean value is calculated. This ratio is used for the calculation of $A$-min for each titration.

The equivalence volume is determined by the Gran function in the absorbance interval 0·6*$A$-max to 0·15*$A$-max. In this interval the main reaction dominates, giving a linear Gran function. The volume is expressed as steps by the stepper motor (one step is equal to approximately 0·02 ml). The result is calculated by dividing the equivalence volume by the volume of the sample, multiplied by the titrant concentration. This means that as long as the same unit is consistently used the unit of the volume is unimportant.

### Results and discussion

The titration set-up described was used on the YMER-80 arctic expedition and found to work well. This expedition investigated sediments, water and air in the region extending from Greenland to Franz Josef's Land (between 78° and 82·5° north). The YMER-80 expedition used the Swedish ice-breaker *YMER* as its base and ran from July to September 1980. One great advantage when working on board a ship is that the sample volume is determined while the titration is being done. About 300 calcium titrations were performed during the 50 days of the YMER expedition. During that time communication between the PET and the external devices never failed. The only problem occurred when the geological winch started, resulting in voltage drops in the power-line.

Ten consecutive titrations of deep water (from 3000 m) performed on board the *YMER* gave the mean value of 10·205 $mM_w$ (mmol/kg sea-water) with a precision, expressed as coefficient of variation, of 0·16%. In practical work the accuracy is set by the standardization of the $Na_4$EGTA. This is done by making a number of titrations of standard sea-water (the standard is supplied by the Institute of Oceanographic Science, Wormley, Godalming, Surrey, UK). The calcium concentration in the standard sea-water has been determined to be 0·5306 mmol $kg^{-1}$ $0/00^{-1}$ [6].

The time from the start of one titration to the beginning of the next is approximately 20 min.

The results reported above show that personal computers used as controlling devices in simple analytical instrumentation can fulfill all the necessary requirements.

### Acknowledgements

The authors are indebted to Professor David Dyrssen and Dr Daniel Jagner for helpful discussions.

### References

1. Betteridge, D. and Goad, T. B., *Analyst*, **106** (1981), 257.

2. Granéli, A. and Anfält, T., *Analytica Chimica Acta*, **91** (1977), 175.

3. Fisher, E. and Jensen, C. W., *PET and the IEEE 488 bus (GPIB)* (Osborne/McGraw-Hill, California, 1980).

4. Jagner, D. *Analytica Chimica Acta*, **68** (1974), 83.

5. Gran, G., *Analyst*, **77** (1952), 661.

6. Anfält, T. and Granéli, A., *Analytica Chimica Acta*, **86** (1976), 13.

### Imperial College Course on Microcomputers in Chemical Instrumentation

A third intensive five-day course on microcomputers in chemical instrumentation will be held from Monday to Friday 20–24 September, at the Chemistry Microprocessor Unit of the Chemistry Department at Imperial College, London. The course is designed for those concerned with data aquisition and the control of instruments in the laboratory, who need to gain first-hand experience of the revolutionary impact of microcomputers on laboratory practice. No previous experience of computing is required. The course includes 12 lectures and seminars, but most of the time is spent gaining 'hands-on' experience in the microcomputing laboratory. All instruction is in BASIC. After a review of the use and economics of microcomputers, the subjects covered include interfacing, choice of programming languages, word processing and the choice of peripherals. The course fee is £300 including lunches, tea and coffee and the course notes (250 pages).

*For details and an application form contact Dr Nick Goddard, Chemistry Microprocessor Unit, Department of Chemistry, Imperial College, London SW7 2AY.*

